# Porphyrins on acid: kinetics of the photoinduced-protonation of tetrakis(4-carboxyphenyl)-porphyrin†

**DOI:** 10.1039/d4cp02542c

**Published:** 2024-09-16

**Authors:** P. Tim Prins, Dorota Rutkowska-Zbik, Sonja Pullen, Bettina Baumgartner

**Affiliations:** a Van’t Hoff Institute for Molecular Sciences, University of Amsterdam Science Park 904 1098 XH Amsterdam The Netherlands b.baumgartner@uva.nl; b Jerzy Haber Institute of Catalysis and Surface Chemistry, Polish Academy of Sciences Niezapominajek 8 30-239 Krakow Poland

## Abstract

Free-base porphyrins can be protonated, which significantly impacts their electronic and excited state properties. While excited state dynamics are well explored for either neutral or fully protonated porphyrins, the intermediate region has not yet been explored, although their potential implications for photocatalytic reactions are evident. This study explores how partial protonation affects the nature and properties of photoexcited states of tetrakis(4-carboxyphenyl)porphyrin (TCPP) using steady-state and nanosecond transient absorption spectroscopy. Global-fit analysis of the decay curves revealed the formation of a protonated excited triplet state from the neutral triplet state, as well as the long lifetimes of these species of up to 120 μs. The photoexcited triplet state of TCPP functions as a photobase, which was confirmed by computational analysis of the electron density of the exited states showing increased nucleophilicity at the unprotonated nitrogen atoms of the porphyrin core. These findings indicate that photoinduced protonated excited triplet states can function as electron acceptors with anodically shifted redox potentials, opening new pathways for porphyrin-based photoreactions.

## Abstract 2

Partial protonation of free-base porphyrins impacts their excited states, with implications for photocatalysis, studied *via* steady-state and nanosecond spectroscopy.

## Graphical abstract

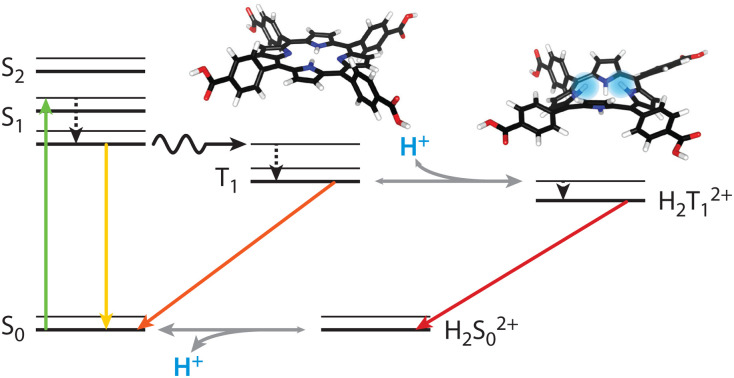

## Introduction

Porphyrins are studied intensively due to their important role in various biological processes and their potential applications in materials science, catalysis, energy conversion, and photodynamic therapy.^1–7^ Their unique optical and electronic properties arise from their conjugated macrocyclic structure, which allows for extensive π-electron delocalization. This structure enables porphyrins to transfer energy as photosensitizers or electrons as photoredox catalysts under light irradiation, facilitating applications such as singlet oxygen generation,^8^ C–C coupling photoredox catalysis,^3^ and CO_2_ photoreduction.^9–12^ Furthermore, porphyrins have been integrated as linkers into metal–organic frameworks (MOFs), where their intrinsic reactivity can be combined with the confinement effect induced by the MOF pores.^8,13,14^

**Fig. 1:**
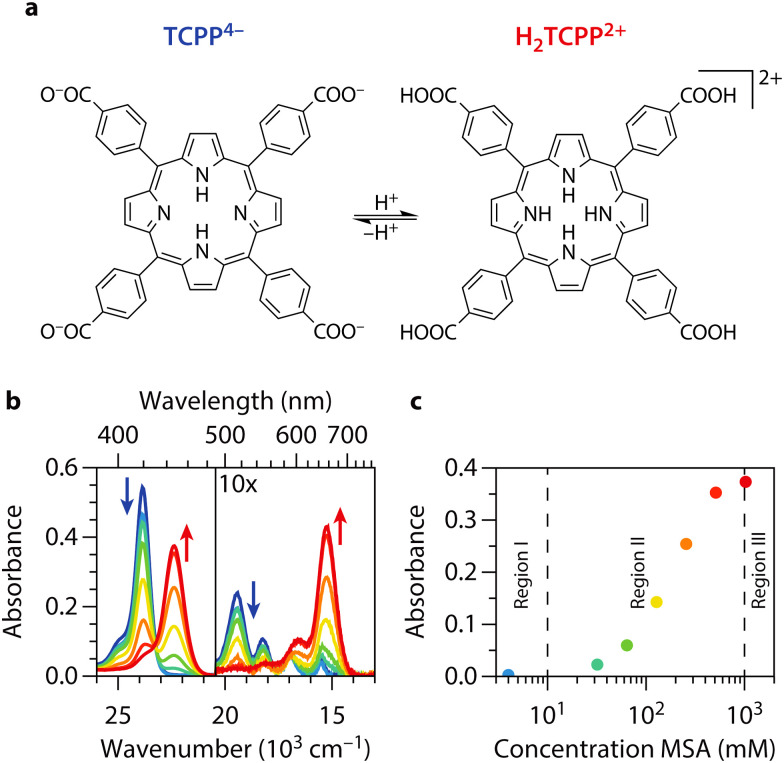
**Steady-state absorption spectroscopy of TCPP and H**_**2**_ **TCPP** ^**2+**^. (a) Molecular structure of carboxylate-form of tetrakis(4-carboxyphenyl)-porphyrin and its fully protonated diacid (TCPP^4−^ and H_2_TCPP^2+^). (b) UV/vis spectra of 1.26 μM TCPP^4−^ in DMF at different acid concentrations from blue to red (0, 4, 32, 64, 128, 256, 512, and 1024 mM MSA). The Soret band (S_0_ to S_2_ transition) is located at 23.9 × 10^3^ cm^−1^ (419 nm) for TCPP and at 22.4 × 10^3^ cm^−1^ (446 nm) for H_2_TCPP^2+^. The region with the four Q-bands of the porphyrin (lower energy section of the plot) is zoomed in 10 times for visualization purposes. H_2_TCPP^2+^ only shows two Q-bands for reasons discussed in the text. (c) Absorbance at 22.4 × 10^3^ cm^−1^, a direct measure to the amount of H_2_TCPP^2+^, as function of MSA concentration. We indicate three regions, neutral (I), intermediately protonated (II), and fully protonated (III).

Recently, we have explored free-base porphyrin-based MOFs, which are known photocatalysts for CO_2_ reduction without the need for an additional co-catalyst.^15,16^ As the role of the porphyrin photocatalyst in this reaction is still unclear, in our previous study we focused on the initial stage of CO_2_ photoreduction, namely the co-adsorption of the reactants CO_2_ and H_2_O within the framework.^15^ Within the confinement of MOF pores, we observed that water interacts with the porphyrin incorporated as a linker into the MOF structure, causing a bending of the initially planar porphyrin molecules. Unlike metalated porphyrins, free-base porphyrins can undergo protonation at the nitrogen atoms within the macrocycle (see Fig. 1(a)). This protonation induces a significant change in the molecular geometry, disrupting the π-system, and causing the porphyrin rings to adopt non-planar conformations.^17^ This finding was surprising as protonation of the homogeneous porphyrin building blocks typically requires the presence of an acid, which was not added during water vapor adsorption. It appeared that simply filling the pores with water in the confinement of the MOF pores was sufficient to partially protonate the porphyrin linker. Increasing acidity in confinement has been observed in microporous materials such as zeolites or MOFs.^18^ Our previous study focused on MOFs with Zr-clusters, which can act as Lewis acid sites, creating Brønsted acid sites upon MOF hydration. Based on this, it was reported for UiO-66 (a Zr-based MOF with terephthalic acid linkers instead of porphyrin linkers) that hydration can lower the external pH of MOF-containing suspensions to as low as 1.6.^19^ As the porphyrin-based MOFs are built from the same Zr-cluster, this shift in pH might explain our findings of partial protonation of the porphyrin linkers with a p*K*_a_ of the core nitrogen atoms of around 1.^20,21^ Given the widespread use of porphyrin-based porous materials in various photocatalytic reactions,^22^ we were surprised that this acidic environment, to the best of our knowledge, has not yet been considered for mechanistic studies on the CO_2_ photoreduction. This prompted us to make the first steps in that direction by investigating how partial protonation affects the photoexcited states of porphyrins.

As current spectroscopic instrumentation to study excited state dynamics of MOFs still requires adjusting of measurement conditions varying from real reaction conditions, *e.g.* using emulsifiers for MOF suspensions,^23–25^ we choose to investigate homogeneous porphyrin linker solutions as a first step. During visible light irradiation, porphyrins are excited from the ground singlet state (S_0_) to higher singlet states S_*n*_, after which they undergo fast vibrational relaxation to S_1_ and high-efficiency intersystem crossing to yield a triplet state (T_1_).^26^ In contrast to neutral free-base porphyrins, protonated or ruffled porphyrins (with sterically demanding geometry, which induces bending) show enhanced S_1_ → T_1_ intersystem crossing and S_1_ → S_0_ internal conversion rates, quenching the singlet fluorescence.^27,28^ Furthermore, reduced triplet lifetimes and redshifted excited triplet state absorption spectra were observed for fully protonated *meso*-arylporphyrin diacids in water.^29–32^ However, insights in the influence of partially protonated *meso*-arylporphyrins on the photoexcited states are still pending.

In this work, we study the excited state dynamics of tetrakis(4-carboxyphenyl)porphyrin (TCPP) as a function of a proton source concentration using steady-state and nanosecond transient absorption spectroscopy. We find that the photoexcited triplet state of TCPP is protonated more easily compared to the ground state and thus acts as photobase. Using global fitting analysis and reference experiments, we established the full overview of the states involved and their reaction rates. The experimentally observed higher basicity of the triplet state is computationally explained by a change in electron density on the unprotonated nitrogen atoms in the porphyrin macrocycle when going from the singlet (ground) state to the triplet state. Protonated porphyrins can function as electron acceptors rather than electron donors, and their redox potentials shift anodically upon protonation.^33–35^ Consequently, we believe that generating photoinduced protonated excited triplet states in the presence of only catalytic concentrations of acid represents a compelling pathway to expand the reaction scope of porphyrinic photoreactions.

## Results

*meso*-Arylporphyrins tend to aggregate in solution, which significantly alters their optical and electronic properties. We choose dimethylformamide (DMF) as solvent to avoid aggregation and accurately assess the excited state dynamics of monomeric tetrakis(4-carboxyphenyl)porphyrin (TCPP).^36^ Additionally, we prepared a series of concentrations to determine the range of linear correlation between concentration and absorbance in the UV/vis steady-state spectra, ensuring monomeric porphyrin solutions. For this reason and further reasons discussed below, we choose to work with a 1.26 μM solution of TCPP in DMF. The steady state UV/vis spectra of TCPP in presence of different concentrations of methane sulfonic acid (MSA) are shown in Fig. 1(b). In DMF, TCPP is present in its COO^−^ form (TCPP^4−^). As the carboxylate groups are more basic, they undergo protonation before the pyrrolic nitrogen atoms in the porphyrin core at MSA concentrations as low as 4 mM.^20^ As the electronic configuration of the porphyrin changes from TCPP^4−^ with COO^−^ moieties to TCPP^0^ with COOH groups upon protonation, we find a slight decrease across the entire absorption spectrum. We will use ‘TCPP’ to refer to both species with unprotonated pyrrolic nitrogen atoms in the porphyrin core (TCPP^4−^ and TCPP^0^). Further addition of MSA, yields the fully protonated diacid (H_2_TCPP^2+^) of TCPP, which shows redshifted steady-state absorption features.^27,28^ When adding MSA > 4 mM, a new band arises at 22.4 × 10^3^ cm^−1^ (Table 1). This feature corresponds to the Soret band (S_0_ to S_2_ transition, *i.e.* B(0,0)) of the diacid (H_2_TCPP^2+^)^29,37^ and appears at the expense of the Soret band of TCPP at 23.9 × 10^3^ cm^−1^ (Table 1). At the same time the four weaker Q-bands (S_0_ to S_1_ transitions, *i.e.* Q_*y*_(1,0), Q_*y*_(0,0), Q_*x*_(1,0), and Q_*x*_(0,0) from high to low energy) decrease in intensity and cluster together in two broader and more intense bands at 16.6 and 15.3 × 10^3^ cm^−1^, respectively Q(1,0) and Q(0,0). This can be explained by considering the origin of the Q-band transitions in TCPP: the Q-band is split into two bands due to vibrational excitations and further split because of a lower symmetry due to NH protons in the porphyrin core. Subsequently, when H_2_TCPP^2+^ is formed, the symmetry is increased (see Fig. 1(a)), and the two vibrational bands remain.^38^ For other porphyrins, monoprotonated species are also observed.^39,40^ However, this is usually not the case for tetraphenyl-based porphyrins. In these compounds, the protonation of both core nitrogen atoms effectively occurs in one step, with the first protonation being the rate-determining step.^33,41^ We also find no indication of a third, mono-protonated species in the spectra presented in Fig. 1(b). Instead, there is a clear isosbestic point between the spectra of TCPP and H_2_TCPP^2+^ at 23.3 × 10^3^ cm^−1^. Additionally, the absence of bands corresponding to H- and J-type aggregates, which would shift the Soret band at 23.9 × 10^3^ cm^−1^ to around 25 × 10^3^ cm^−1^ and 20 × 10^3^ cm^−1^ respectively, indicates that aggregates are not formed.^37,42^

**Table 1:** Steady-state UV/vis band assignments and positions in cm^−1^ (nm)

TCPP	B(0,0)	Q_*y*_(1,0)	Q_*y*_(0,0)	Q_*x*_(1,0)	Q_*x*_(0,0)
	23.9 (419)	19.4 (515)	18.2 (548)	16.9 (591)	15.5 (645)
H_2_TCPP^2+^	B(0,0)	—	—	Q(1,0)	Q(0,0)
	22.4 (447)	—	—	16.6 (602)	15.3 (655)
*Δ*	−1.5 (28)	—	—	−0.3 (11)	−0.2 (10)

Fig. 1(c) shows the absorbance at the H_2_TCPP^2+^ Soret band as function of MSA concentration. Three distinct regions are indicated in this figure. The first region corresponds to neutral TCPP^0^ with only the carboxylic acid groups protonated. The second region shows the coexistence of both TCPP^0^ and H_2_TCPP^2+^. The third region represents the presence of only H_2_TCPP^2+^. These regions will be referenced in subsequent discussions.

**Fig. 2:**
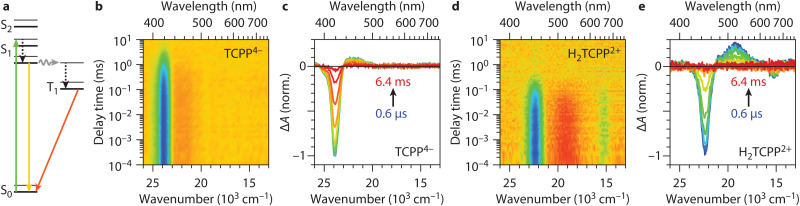
**Excited state dynamics of TCPP** ^**4−**^ **and H**_**2**_ **TCPP** ^**2+**^. (a) Jablonski diagram for TCPP neutral environment (region I), showing the excitation into the singlet (green arrow), vibrational relaxation (black arrow), singlet relaxation (yellow arrow), intersystem crossing (grey arrow), and triplet relaxation (orange arrow) pathways. The same applies to H_2_TCPP^2+^, *i.e.* fully protonated TCPP (region III), except that it does not have a split S_1_ level (see text). (b) and (d) Transient absorption heatmaps on TCPP^4−^ and H_2_TCPP^2+^ after excitation at respectively 515 nm and 665 nm. (c) and (e) Spectral slices of the heatmap at delay times 0.6 μs, 3.0 μs, 9.1 μs, 24 μs, 63 μs, 160 μs, 0.4 ms, 1.0 ms, 2.5 ms, and 6.4 ms from blue to red. The bleach positions correspond to the Soret bands in the steady state spectra (Fig. 1(b)). When fitting a single exponential to the bleach intensity time traces, lifetimes of 1.2 ms and 120 μs are obtained for TCPP^4−^ and H_2_TCPP^2+^ respectively.

To investigate the excited state dynamics in the three stages of protonation we use transient absorption (TA) spectroscopy. In short, the in-house assembled nanosecond TA setup^43^ used in this work is composed of a tuneable nanosecond Nd:YAG-laser system as pump, a xenon flash lamp as probe, a gated intensified CCD camera coupled to a spectrograph to measure the probe spectra, and digital delay generators to change the delay time of the probe together with the gate of the camera with respect to the pump (for more details, see the Experimental section). To avoid quenching of the T_1_ by singlet oxygen formation (^3^O_2_ to ^1^O_2_),^44^ we purged our samples with argon for 30 minutes. Additionally, we verified that the TCPP^4−^ concentration is low enough to prevent interaction between porphyrin molecules (*e.g.* aggregation). A concentration of 1.26 μM resulted in a saturation of the T_1_ lifetime around 1.5 ms (Fig. S1, ESI†).^45^ At higher concentrations there is an increased chance that molecules in a T_1_ state collide and that T_1_–T_1_ annihilation occurs, shortening the T_1_ lifetime.^46^ To minimize the amount of excess energy we excite into the Q_*y*_(1,0) band at 515 nm for TCPP and at Q(0,0) band at 665 nm for H_2_TCPP^2+^ and not in the stronger absorbing Soret band. It is well known that within nanoseconds high-efficiency intersystem crossing from the singlet (S_1_) into the triplet state (T_1_) occurs. The subsequent T_1_ is of high interest for photocatalytic reactions because it has a lifetime of milliseconds since the transition back to the ground state (S_0_) is spin forbidden.^45^Fig. 2(a) shows the Jablonski diagram for the excited state dynamics of TCPP.

Fig. 2(b)–(e) shows the full TA heatmaps and differential spectra at different delay times of TCPP^4−^ and H_2_TCPP^2+^ respectively. Note that TCPP^0^, obtained at 4 mM MSA concentration, as discussed above, shows identical heatmaps as TCPP^4−^ (Fig. S2, ESI†). The spectra show a negative band, *i.e.* less absorbance, at their respective Soret positions (see Fig. 1(b)). This ground-state bleach is due to population of the ππ* states and subsequently T_1_.^47^ They also show a weaker positive feature, *i.e.* more absorbance, originating from excited state absorbance of T_1_ to higher triplet levels (T_*n*_), also known as photoinduced absorbance. When fitting the decay of the bleach with a single exponential, we find lifetimes of 1.2 ms, 1.0 ms, and 120 μs for TCPP^4−^, TCPP^0^, and H_2_TCPP^2+^, respectively. Furthermore, we found that the H_2_TCPP^2+^ triplet lifetime, obtained when exciting the samples at 665 nm, does not show any dependence on the MSA concentration in the intermediate region (II) and the fully protonated region (III) (Fig. S3, ESI†). Upon exposure to air, the lifetimes decreased to the sub-μs range, indicating fast quenching of the T_1_ by ^3^O_2_, which can subsequently generate singlet oxygen. This suggests that the triplet states of TCPP^4−^, TCPP^0^, and H_2_TCPP^2+^ have energies greater than 7.9 × 10^3^ cm^−1^ (94 kJ).^48,49^

**Fig. 3:**
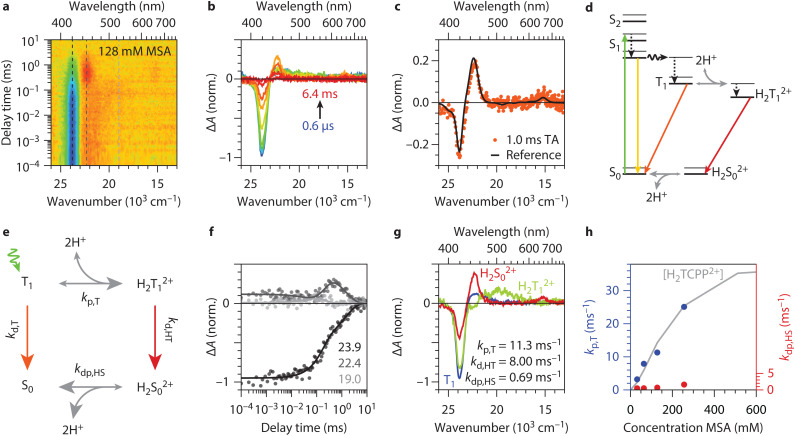
**Excited state dynamics of TCPP in the intermediate concentration range**. (a) Transient absorption (TA) heatmap for TCPP (excitation into Q_*y*_(1,0) at 515 nm) in an acidic environment (128 mM MSA). The photoinduced absorption (red area) around 1 ms is absent in the measurements presented before. (b) Spectral slices of the heatmap at delay times 0.6 μs, 3.0 μs, 9.1 μs, 24 μs, 63 μs, 160 μs, 0.4 ms, 1.0 ms, 2.5 ms, and 6.4 ms from blue to red. The photoinduced absorption is visible in the orange spectra and located at 22.4 × 10^3^ cm^−1^ (446 nm). (c) The TA spectrum at 1.0 ms, same as in panel b, overlapped with in black the difference between the steady-state UV/vis spectrum of 4 mM and 1024 mM presented in Fig. 1(c) scaled arbitrarily. The good overlap between the two proves that the final state in the TA measurements is a photoinduced protonated ground state. (d) Altered Jablonski diagram for TCPP in acidic environment but not fully protonated (region II). (e) Proposed excited-state relaxation and (de)protonation dynamics used for the global fit analysis in panel e, f (see ESI,† S2 for rate equations). (f) Time trace slices of the heatmap at energies 23.9, 22.4, and 19.0 × 10^3^ cm^−1^, like indicated in the heatmap in panel a, with the global fit results (lines). (g) The three spectra of the three states in the global fit model. The three corresponding rates are 11.3, 8.0, and 0.69 ms^−1^. They compare well with separately collected spectra (see Fig. S6, ESI†). (h) The protonation (*k*_p,T_ in red) and deprotonation (*k*_dp,HS_ in blue) rates obtained by global fitting the transient absorption data on the MSA concentrations 32, 64, 128, and 256 mM. The grey line shows the H_2_TCPP^2+^ concentration obtained from steady-state UV/vis also shown in Fig. 1(c) and is scaled arbitrarily.

Fig. 3 shows the excited state dynamics in the intermediate MSA concentration range (II). Fig. 3(a) presents the TA heatmap obtained for partially protonated TCPP/H_2_TCPP^2+^ mixtures, with excitation into the Q_*y*_(0,0) band of TCPP at 515 nm in an acidic environment (128 mM MSA) (Fig. S4, ESI† for other MSA concentrations). This excitation wavelength was chosen, as there is no overlap with bands of H_2_TCPP^2+^, thus allowing sole excitation of TCPP. Comparing Fig. 2(b) with Fig. 3(a), an additional photoinduced absorption band (red) around 1 ms and 22.4 × 10^3^ cm^−1^ is noticeable in the partially protonated sample. Note that TA data after excitation at 665 nm does not show any MSA concentration dependence and solely showed features as obtained in the fully protonated case (Fig. S3, ESI†). Fig. 3(b) shows spectral slices of the heatmap at various delay times that clearly show the photoinduced absorption at 22.4 × 10^3^ cm^−1^ (446 nm) as function of MSA concentration. The spectrum at 1.0 ms, shown in Fig. 3(c), matches the difference between the steady-state UV/vis spectra of 4 mM and 1024 mM MSA in Fig. 1(c). This strong overlap confirms that the final state in the TA measurements is a photoinduced protonated ground state (H_2_S_0_^2+^). Since no protonated H_2_S_0_^2+^ was formed in the presence of oxygen, which quenches T_1_, we confirmed that the pathway proceeds *via* T_1_ (see Fig. S5, ESI†). This allowed us to draw the full picture of the excited state and (de)protonation dynamics, as outlined in Fig. 3(d): similar to the MSA-free dynamics, TCPP is initially excited from the ground state to the singlet excited states, followed by a quick intersystem crossing to T_1_. However, at this point, the dynamics deviate from those shown in Fig. 2(a), as T_1_ undergoes protonation to form H_2_T_1_^2+^, which relaxes back into a protonated ground state H_2_S_0_^2+^. Due to the dynamic acid-dissociation equilibrium, H_2_S_0_^2+^ will release the captured protons, closing the Förster cycle and yielding unprotonated S_0_. A similar behaviour has been reported for tetrakis(4-sulphonatophenyl)porphyrin (TPPS) in water.^50^

We applied the proposed excited state relaxation and (de)protonation dynamics to our transient absorption dataset by means of a global fitting procedure. Fig. 3(e) shows the rate model used for the global fitting. Here, the singlet dynamics and the intersystem crossing are omitted for simplicity and because they do not play a role in the photoinduced protonation as discussed above. The exact rate equations are discussed in ESI S2.† From the data shown in Fig. 2 and Fig. S2 (ESI†), the triplet decay rates of TCPP and H_2_TCPP^2+^ are obtained as *k*_d,T_ = 1 ms^−1^ and *k*_d,HT_ = 8 ms^−1^. This leaves the triplet protonation rate *k*_p,T_ and the ground state (H_2_S_0_^2+^) deprotonation rate *k*_dp,HS_ as the only rates being optimized in the global fitting procedure.

The global fit yielded a successful match, as shown in the great overlap of experimental data with fitted values for time slices at three prominent spectral regions (Fig. 3(f)). This allowed us to retrieve the protonation rate of the T_1_ and the deprotonation rate of H_2_S_0_^2+^, *k*_p,T_ = 11.3 ms^−1^ and *k*_dp,HS_ = 0.69 ms^−1^ for 128 mM MSA concentration. The obtained spectra of the T_1_, H_2_T_1_^2+^, and H_2_S_0_^2+^ from the global fit (Fig. 3(g)) compare well to the separately recorded spectra of T_1_ (Fig. 2(c) and Fig. S2b, ESI†), H_2_T_1_^2+^ (Fig. 2(e) and Fig. S2d, ESI† expect that the bleach is at a different position), and the difference in steady state between S_0_ and H_2_S_0_^2+^ (black curve in Fig. 3(c)). A direct comparison of these spectra is shown in Fig. S6 (ESI†) and underlines our proposed relaxation dynamics in Fig. 3(e). Our findings align with similar slow protonation rates observed for tetraphenylporphyrin (TPP) in DMSO–water mixtures using temperature jump experiments.^33^ The protonation rate is an order of magnitude higher than the deprotonation rate, leading to a considerable net build-up of H_2_S_0_^2+^ at higher MSA concentrations as observed in Fig. 3(b). The slow rates also correlate with long lifetimes for H_2_T_1_^2+^ of 120 μs, crucial for their use in chemical (redox) reactions.

We extended the global-fit analysis to data sets with varying MSA concentrations (Fig. S4, ESI†) to establish the relationship between proton source concentration and (de)protonation rates (Fig. 3(h)). The protonation rate follows the increase in H_2_TCPP^2+^ concentration found in the steady-state UV/vis measurements (Fig. 3(h) grey, same as Fig. 1(c)) and we find a linear dependence of the protonation rate on the MSA concentration ranging from 3.2 ms^−1^ (32 mM MSA) to 25.1 ms^−1^ (256 mM MSA), shown in Fig. 3(h). This indicates first-order kinetics and a reaction-limited protonation rate of 10^5^ M^−1^ s^−1^, as diffusion rates are typically much higher (10^10^ M^−1^ s^−1^). As discussed earlier, this protonation rate corresponds to the addition of the first proton, which induces the structural changes and is the rate-determining step in the protonation process.^39^ These slower rates contrast with those of other aromatic amines, suggesting that structural changes during protonation are responsible for the slower kinetics.^51,52^

In contrast, we find minor dependence of the deprotonation rate of the MSA concentration (ranging from 0.48 to 1.61 ms^−1^, see Fig. 3(h) blue). This suggests that other factors, such as the stabilization of H_2_TCPP^2+^ in DMF or the abstraction of the first proton and reversing the protonation-induced porphyrin deformation, play a more significant role in determining the deprotonation rate.

To support our experimental findings, we conducted Density Functional Theory (DFT) calculations of TCPP and H_2_TCPP^2+^. For all structure optimizations and electronic structure calculations, we utilized the Gaussian 09 program. Fig. S7 (ESI†) presents the obtained optimized geometries of the neutral ground state S_0_ and first triplet excited state T_1_. The dihedral angles of the porphyrin core increased only slightly when transitioning from S_0_ to T_1_ geometry, consistent with previous experimental and computational findings.^53,54^ This observation contrasts with the structural changes observed upon protonation. As reported previously,^35,38^ we find strong ruffling of the tetrapyrrolic unit in the protonated S_0_, which is even more pronounced in the protonated T_1_ geometry (Fig. S8, ESI†). The ground-state findings align with our steady state UV/vis spectra, which showed a redshift of the Soret band due to the disruption of the π-system caused by ruffling (Fig. 1(b)). We propose that the reduced triplet state lifetime observed upon excitation of protonated ground-state molecules is a result of the changed geometry (compare time traces in Fig. S2, ESI†).

**Fig. 4:**
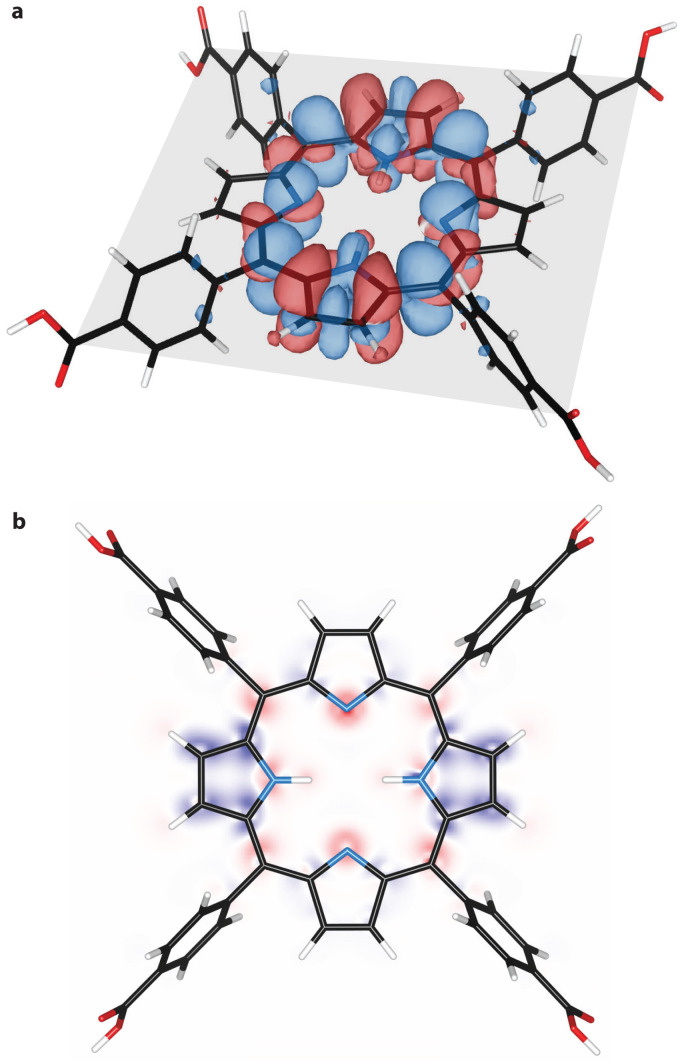
**Differential electron density of TCPP**. (a) Difference in electron density (increased electron density in red, decreased density in blue) between ground state S_0_ and excited triple state T_1_. The porphyrin plane is indicated in grey. (b) Differential electron density in the porphyrin plane. Red indicates an increase and blue a decrease in electron density.

From natural population analysis, we derived the charges of the pyrrolic nitrogen atoms (see Table S1 and Fig. S9, ESI†). Here, a higher uniformity between the charges of the pyrrolic nitrogen atoms at T_1_ was observed. Furthermore, electron density difference maps were derived for the ground state and the triplet state (see Fig. 4(a)). These maps revealed increased electron density, and thus nucleophilicity, at the unprotonated nitrogen atoms in the porphyrin plane. This becomes particularly evident when presenting the differential density maps between T_1_ and S_0_ solely in the porphyrin plane as shown in Fig. 4(b). This map shows increased electronegativity at the unprotonated nitrogen atoms (red cloud) and explains the increased basicity of the first excited triplet state, as derived from transient absorption spectroscopy experiments (Fig. 3). This finding is consistent with the presence of a free lone-pair orbital that binds the hydrogen atom upon protonation. The electron density decreased above and below the porphyrin plane and corresponds to the p-orbitals, which are not involved in the protonation. Lastly, we noted that the nucleophilicity of T_1_ seems to be minimally affected by the solvent DMF (see Fig. S10, ESI†).

## Conclusion

Through steady-state and transient absorption spectroscopy we established that the photoexcited triplet state of TCPP exhibits higher basicity compared to its ground state, effectively acting as a photobase. These findings are supported by DFT calculations, which reveal structural changes upon protonation and highlight the increased nucleophilicity of the first excited triplet state (T_1_). Global-fit analysis revealed slow (de)protonation rates and a reaction-limited protonation step. The next step is to compare these findings with processes in the confined space of MOFs, which requires novel instrumentation that overcomes the strong light scattering of the micrometer-sized MOF crystals.

Our results provide a further step towards elucidating the reaction pathway of CO_2_ photoreduction in porphyrinic porous materials. To the best of our knowledge, neither the high acidity induced by the interaction of water with the Lewis acidic Zr-cluster nor the altered excited state kinetics have been considered. The acidic environment in the pores might influence the reaction pathways by stabilizing protonated species and facilitating proton-coupled electron transfer processes, both important intermediate steps in CO_2_ photoreduction. The excited state redox potentials also play a significant role. It remains to be proven if the excited state redox potentials of porphyrins shift anodically within the MOF environment as well, potentially enabling them to function as electron acceptors rather than donors. Lastly, the long lifetimes of the protonated excited state (up to 120 μs), which we found in this study, confirm their potential as reaction partners. However, the elucidation of the entire CO_2_ photoreduction pathway is beyond the scope of this work. The above-mentioned factors will be the focus of future studies.

Beyond CO_2_ photoreduction, the probable shift in redox behaviour opens exciting possibilities for controlling redox potentials and proton transfer reactions. If photoinduced protonation occurs within the confined environment of MOFs solely in the presence of water, it might drive otherwise unfavourable redox reactions and significantly expand the reaction scope of porphyrinic photoreactions.

## Experimental section

### Porphyrin solutions

1.26 μM TCPP (Porphychem, >98%) was used as is and solutions in DMF (Fisher Scientific, 99.8%) were prepared from 0.1 mg mL^−1^ stock solutions. Methane sulfonic acid (MSA, TCI Chemicals, >99.0%) was dissolved in DMF and added to the TCPP solutions prior the experiments. 2 mL of the solutions were transferred into screw-cap cuvettes (10 × 10 mm, Helma) with septum, further wrapped with parafilm and bubbled with Argon for 30 min prior TA measurements.

### Steady-state UV/vis spectroscopy

Prior and after nanosecond TA experiments steady-state UV/vis spectra were recorded on a Shimadzu UV-2700 spectrometer. Spectra were recorded with 1 nm steps between 350 and 800 nm.

### Nanosecond transient absorption spectroscopy

Nanosecond transient absorptions were recorded with an in-house assembled setup.^43^ The excitation wavelength was generated using a tuneable Nd:YAG-laser system (NT342B, Ekspla). The laser system was operated at a repetition rate of 10 Hz with a pulse length of 5 ns. The probe light was operated at 20 Hz and was generated by a high-stability short arc xenon flash lamp (FX-1160, Excelitas Technologies), using a modified PS302 controller (EG&G). Using a 50/50 beam splitter, the probe light was split equally into a signal beam and a reference beam and focused (bi-convex lens 75 mm) on the entrance slit of a spectrograph (SpectraPro-150, Princeton Instruments) with a grating of 150 g mm^−1^, blazed at 500 nm. The probe beam (*A* = 1 mm^2^) was passed through the sample cell and orthogonally overlapped with the excitation beam on a 1 mm × 10 mm area. The excitation energy was recorded by measuring the excitation power at the back of an empty sample holder and was reduced using a variable attenuator, which contains a half-waveplate and a beamsplitter, to a pulse energy of 0.14 mJ for 515 nm and 0.07 mJ for 665 nm. To correct for fluctuations in the flash lamp spectral intensity, the reference was used to normalize the signal. The probe and reference beams were simultaneously recorded with a single gated, intensified CCD camera (PI-MAX3, Princeton Instruments), which features an adjustable gate with a minimum setting of 2.9 ns. A gate setting of 50 ns, along with software binning, was used to improve the dynamic range and signal-to-noise ratio. Two delay generators (DG535 and DG645, Stanford Research Systems) were used to trigger the excitation and to change the delay of the flash lamp together with the gate of the camera during the experiment. The setup was controlled by a LabVIEW program written in-house. The order of the delay times at which the transient spectra were measured was randomized and the absorption of the sample was monitored during the experiment. At every delay time 100 spectra were collected and subsequently averaged for a good signal-to-noise ratio.

### Data processing

Data processing was performed in Mathematica. The global fitting was also performed in Mathematica *via* a self-written least-squares fitting procedure.

### Computational characterization

The geometries of the reported structures were obtained within Density Functional Theory (DFT) using B3LYP functional^55–59^ and 6-31G(d) basis set.^60,61^ The influence of solvent was accounted by polarizable continuum model (PCM), using the integral equation formalism variant (IEFPCM). All calculations were done in Gaussian 09 program.^62^

## Author contributions

PTP: conceptualization, data curation, investigation, visualization, validation, formal analysis, writing – original draft; DRZ: methodology, formal analysis, investigation, writing – review & editing; SP: methodology, writing – review & editing; BB: investigation, writing – original draft, conceptualization, validation, funding acquisition, supervision.

## Data availability

The data supporting this article has been included as part of the ESI.† Raw and processed data for this article, including steady-state and transient absorption spectra, as well as optimized porphyrin geometries are available at zenodo.org under the manuscript title.

## Conflicts of interest

There are no conflicts to declare.

## Supplementary Material

CP-026-D4CP02542C-s001

CP-026-D4CP02542C-s002

CP-026-D4CP02542C-s003

CP-026-D4CP02542C-s004

CP-026-D4CP02542C-s005
