# Supplementary material for: Porphyrins on acid: kinetics of the photoinduced-protonation of tetrakis(4-carboxyphenyl)-porphyrin
Source: Phys Chem Chem Phys. 2024 Sep 16;26(37):24524–32. doi: 10.1039/d4cp02542c (PMC11403973; doi:10.1039/d4cp02542c)
Supplement: CP-026-D4CP02542C-s003 [file CP-026-D4CP02542C-s003.pdf]

Supplementary Information (SI) for Physical Chemistry Chemical Physics.
This journal is © the Owner Societies 2024

**Supporting Information for**

# Porphyrins on Acid: Kinetics of the Photoinduced-Protonation of tetrakis(4-carboxyphenyl)-porphyrin

P. Tim Prins,[1] Dorota Rutkowska-Zbik,[2] Sonja Pullen,[1] Bettina Baumgartner[1,*]

[1]Van't Hoff Institute for Molecular Sciences, University of Amsterdam, Amsterdam, the Netherlands

[2]Jerzy Haber Institute of Catalysis and Surface Chemistry, Polish Academy of Sciences, Niezapominajek 8, 30-239 Krakow, Poland

[*]Email: b.baumgartner@uva.nl

## Table of Contents

S1. Transient absorption spectroscopy .......... 2
S2. Rate equation model for global fit .......... 7
S3. Computational chemistry .......... 8
References .......... 10

## S1. Transient absorption spectroscopy

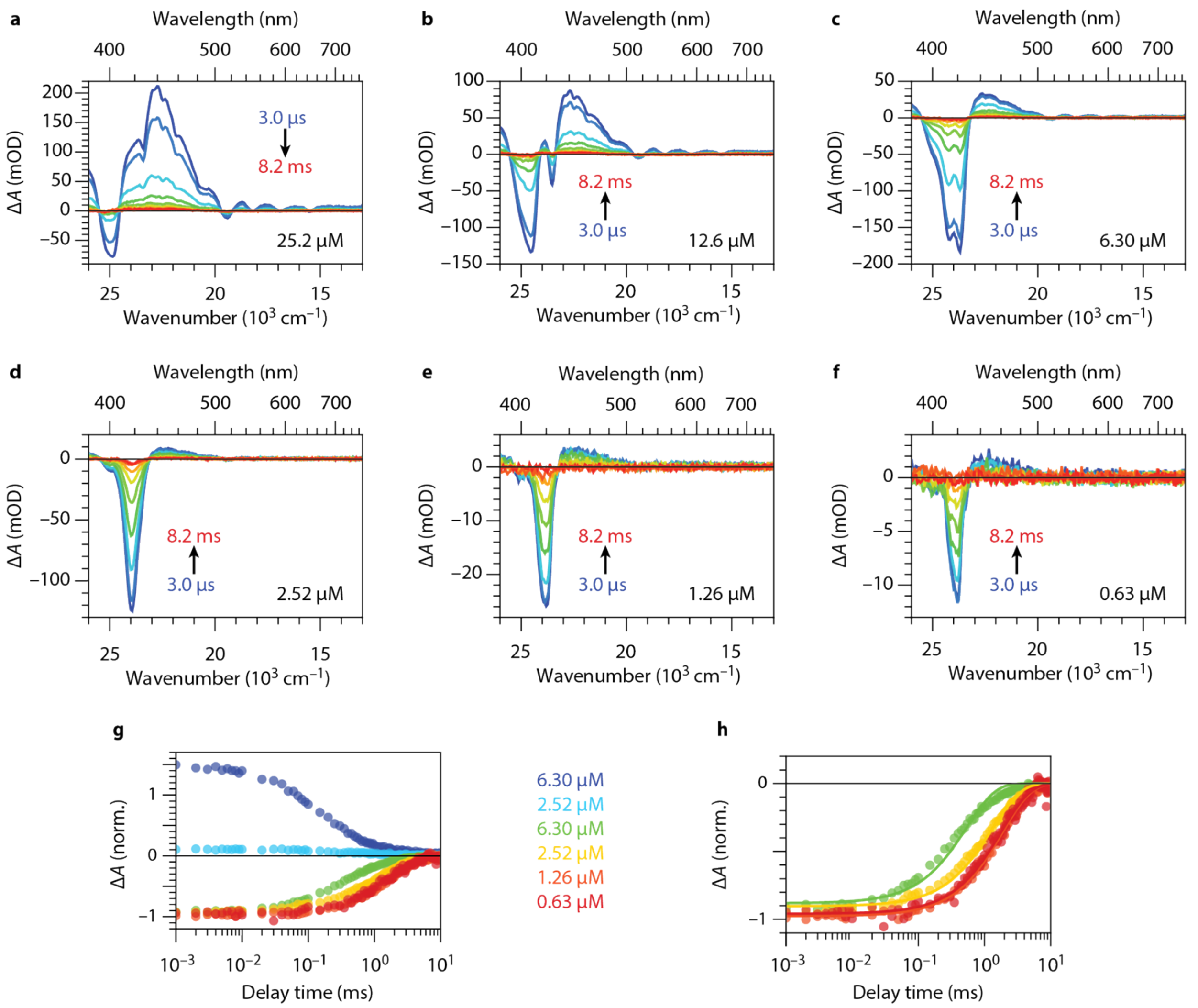


**Figure S1. Transient absorption spectroscopy for different concentrations of $TCPP^{4-}$ in DMF.** (**a–f**) Transient absorption spectra for $TCPP^{4-}$ concentrations 25.2, 12.6, 6.30, 2.52, 1.26, and 0.63 μM at delay times 3 μs, 40 μs, 300 μs, 800 μs, 1.6 ms, 2.6 ms, 4.2 ms, 6.2 ms and 8.2 ms (from blue to red). (**g**) The time trace at $23.9 \cdot 10^3$ $cm^{-1}$, *i.e.* the position of the Soret band, for the different concentrations from high (blue) to low (red) concentration. (**h**) Single exponential fit of the data shown in **g** for the lowest four concentrations. The fitted lifetimes values are 0.54 ms, 1.3 ms, 1.9 ms and, 1.8 ms. This indicates that the lifetime saturates when the concentration is 1.26 μM or lower. 1.26 μM is the concentration used at all the other experiments presented in this work.

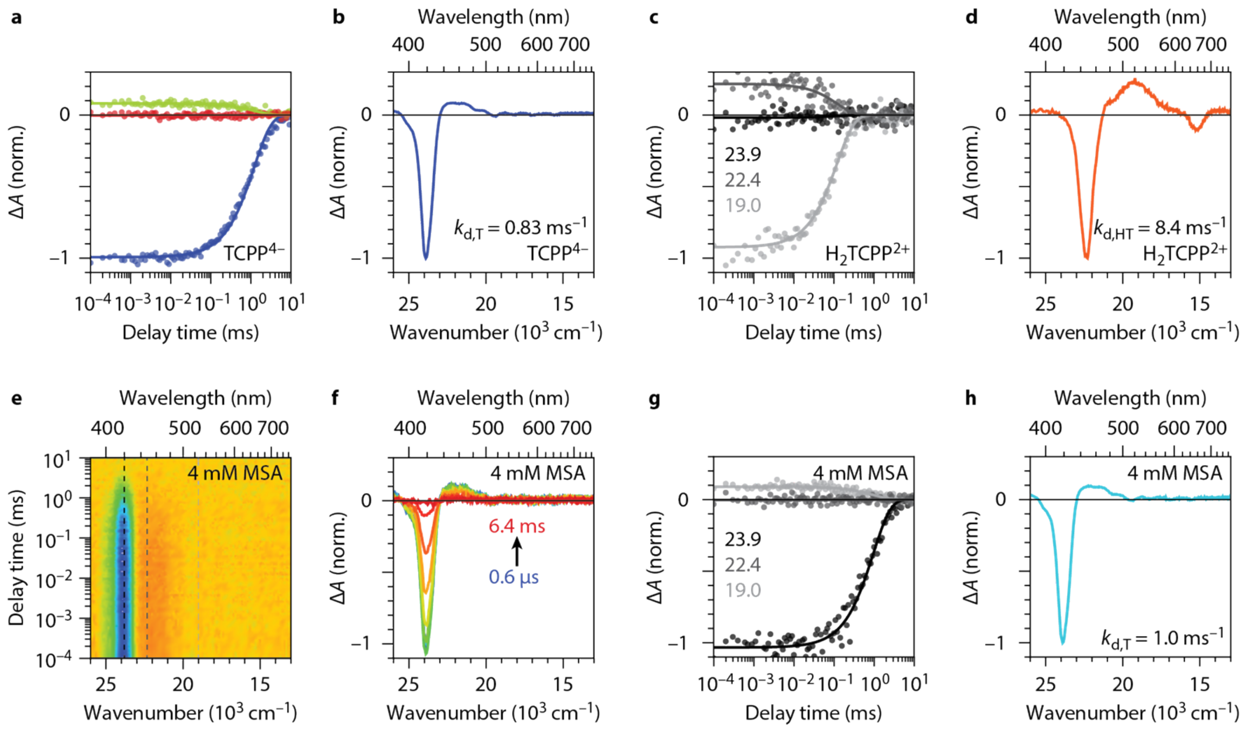


**Figure S2. Additional figures for $TCPP^{4-}$, $TCPP^0$, and $H_2TCPP^{2+}$.** (**a–d**) Global fit analysis on $TCPP^{4-}$ and $H_2TCPP^{2+}$ (data shown in **Figure 2**), showing the time trace in **a** and **c** at the energies 23.9, 22.4, and $19.0 \cdot 10^3$ $cm^{-1}$, and the spectrum and decay rate obtained from the global fitting procedure in **b** and **d** respectively. (**e–h**) Data obtained on TCPP in 4 mM MSA ($TCPP^0$). The panels show (**e**) transient absorption heatmap, (**f**) spectral slices at the same delay times as in **Figure 2**, (**g**) time trace with global fit result at the same energies as panel a,c, (**h**) the spectrum obtained from the global fitting procedure and the decay rate. The spectra in panel b and h are almost identical and the decay rates are also similar. This implies that protonation of the carboxy groups has little effect on the transient absorption measurements as further discussed in the main text.

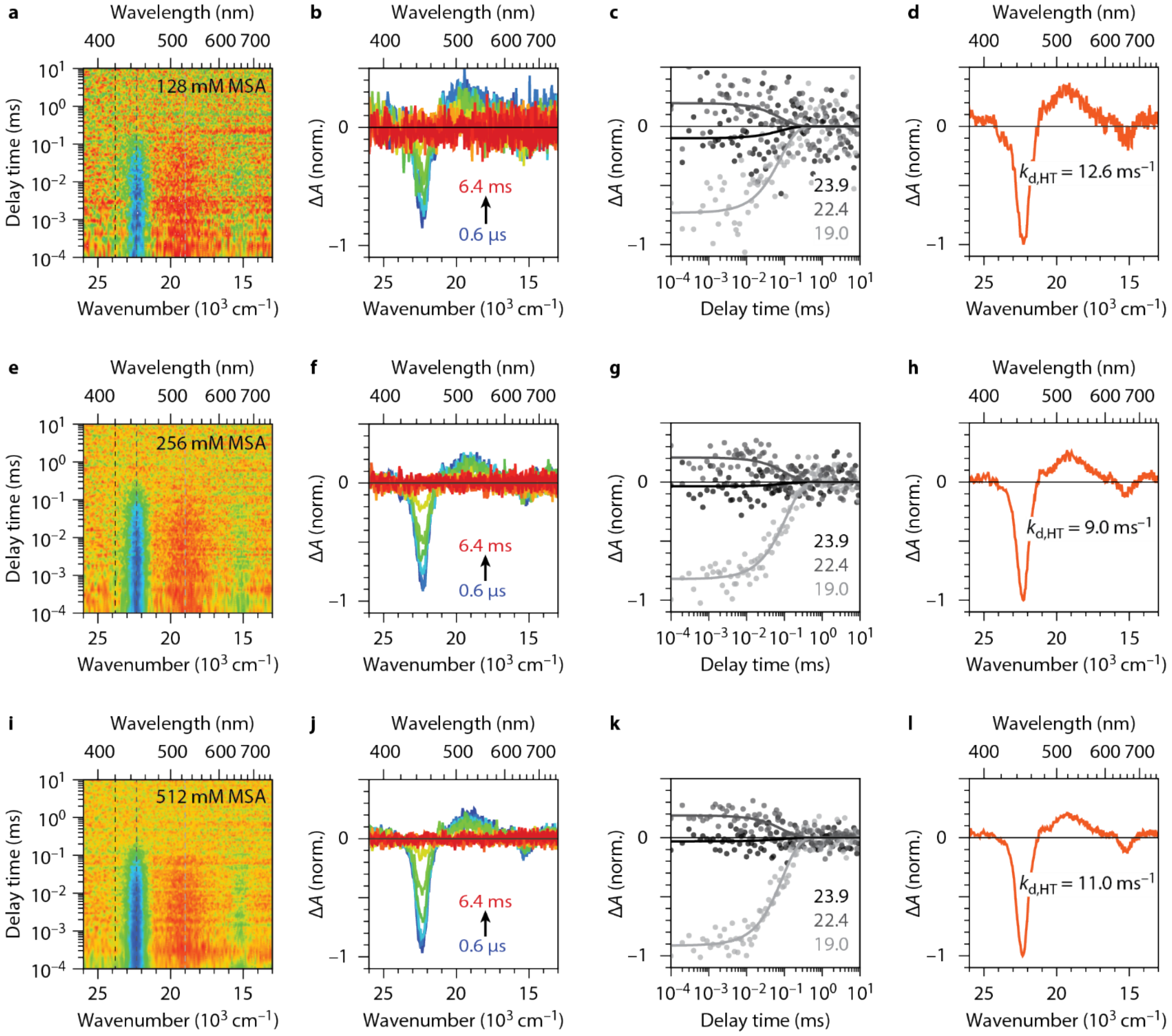


**Figure S3. $H_2TCPP^{2+}$ triplet ($k_{d,HT}$) lifetimes in different MSA concentrations (128, 256, and 512 mM MSA) after excitation in the Q(0,0) band with 665 nm light.** Heatmaps, spectral slices at 0.6 µs, 3.0 µs, 9.1 µs, 24 µs, 63 µs, 160 µs, 1.0 ms, 2.5 ms, and 6.4 ms from blue to red, time trace at the energies 23.9, 22.4, and 19.0·$10^3$ $cm^{-1}$ with global fit result (lines), and global fit results for 128 mM MSA (**a–d**), 256 mM MSA (**e–h**), and 512 mM MSA (**i–l**).

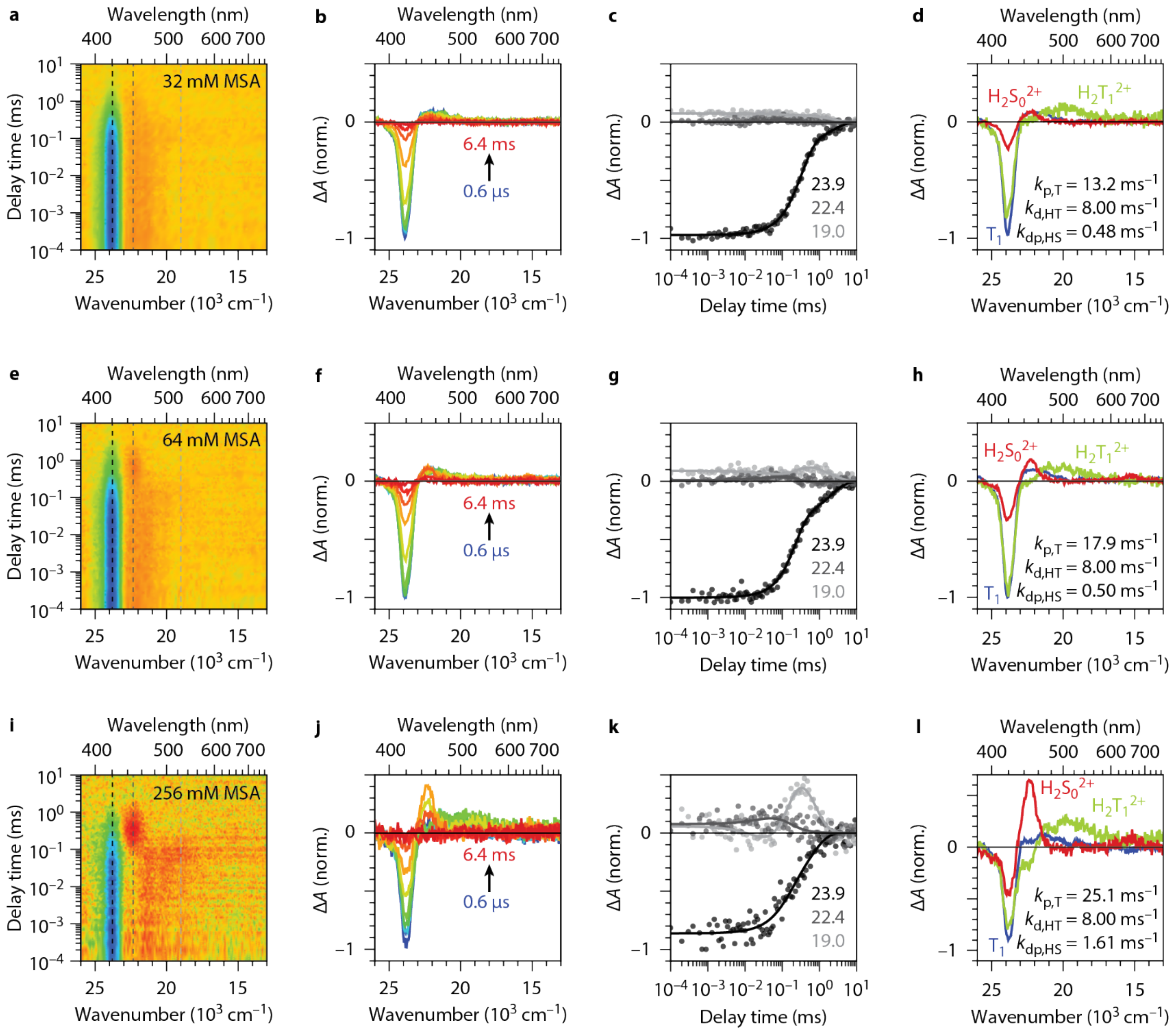


**Figure S4. Transient absorption spectroscopy heatmaps of TCPP in DMF with increasing amount of MSA.** MSA concentrations are 32 mM (**a-d**), 64 mM (**e-h**), and 256 mM (**i-l**). The data for 128 mM MSA is discussed in the main text. The PIA absorption feature around 1 ms and 22.4·10$^3$ cm$^{-1}$ increases with MSA concentration. See the main text for further discussion of this data.

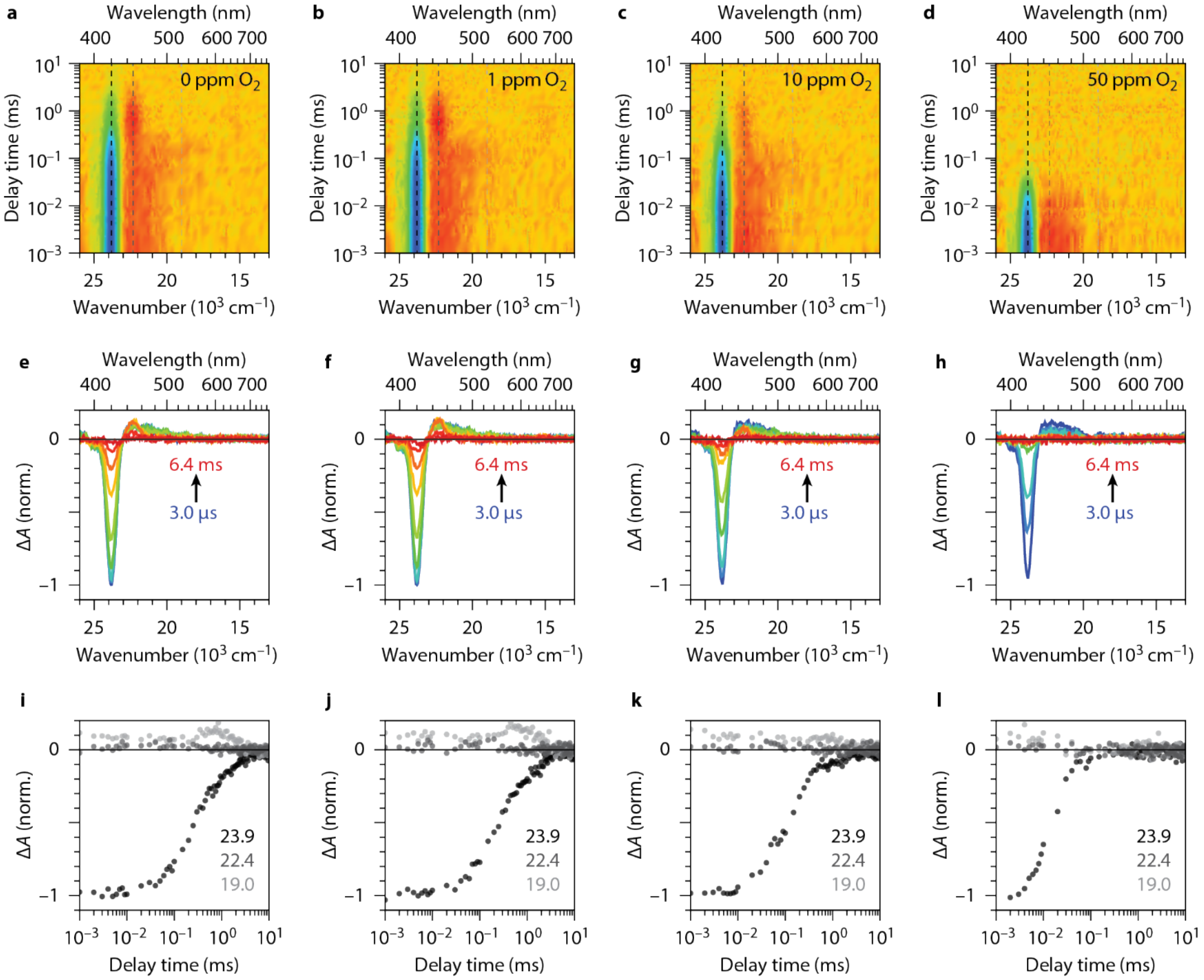


**Figure S5. The effect of oxygen on the excited state dynamics of TCPP in 100 mM MSA (region II).** Transient absorption heatmaps for 0 (**a**), 1 (**b**), 10 (**c**), and 50 ppm oxygen (**d**). Spectral slices at delay times 3 µs, 11 µs, 22 µs, 60 µs, 160 µs, 0.4 ms, 1.0 ms, 2.5 ms, and 6.4 ms for 0 (**e**), 1 (**f**), 10 (**g**), and 50 ppm oxygen (**h**). Time traces at energies 23.9, 22.4, and 19.0·$10^3$ $cm^{-1}$ like indicated in the heatmaps for 0 (**i**), 1 (**j**), 10 (**k**), and 50 ppm oxygen (**l**). The PIA feature observed in the heatmaps around 1 ms and 22.4·$10^3$ $cm^{-1}$, in the orange spectra, and in the light grey time traces disappears with increasing oxygen amount. For this reason, it can be concluded that the photo-induced protonated ground state is a result from protonation of the triplet ($T_1$) and not of a single ($S_1$) state. Since only the triplet can be quenched by oxygen by generating ${}^1O_2$.[1] If the triplet is quenched and the protonation rate remains the same, there will not be any protonation if the quenching rate is much higher than the protonation rate. See main text for further discussion.

*Method.* The different oxygen amounts are prepared by mixing deaerated TCPP in 100 mM MSA in DMF (by bubbling with argon) with aerated (left open at atmosphere at room temperature) TCPP in 100 mM MSA in DMF. The solubility of oxygen in DMF at atmospheric pressure and room temperature is 3.89·$10^{-4}$ (mole fraction), which gives 389 ppm oxygen in DMF.[2] The volumes are adjusted accordingly to give 1, 10, and 50 ppm oxygen in the samples.

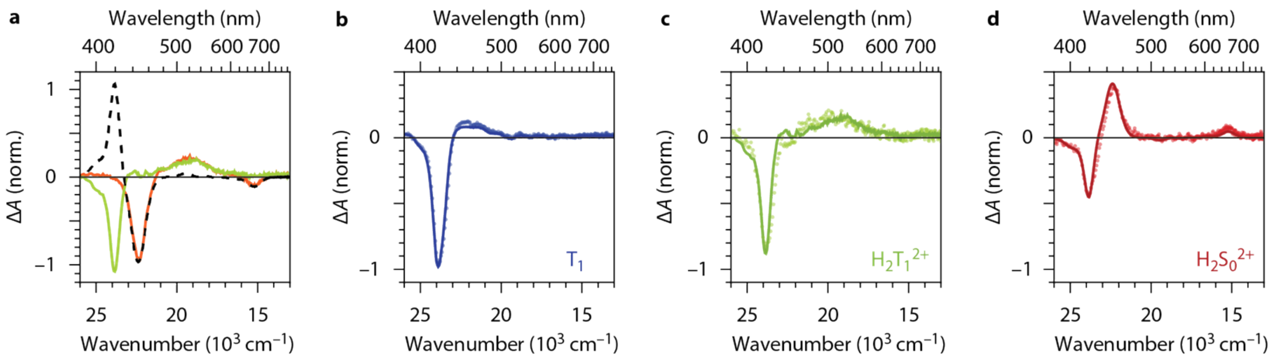


**Figure S6. Comparison of the spectra obtained from global fitting data obtained from the experiment with 128 mM MSA concentration shown in Figure 3 with reference measurements.** (**a**) Reconstruction of a protonated TCPP $H_2T_1^{2+}$ spectrum originating from excitation into a non-protonated $S_0$ molecule, *i.e.* bleach at 23.9·10$^3$ cm$^{-1}$ (green). In orange the $H_2T_1^{2+}$ spectrum obtained after excitation of a $H_2S_0^{2+}$ molecule (Figure S2d), *i.e.* bleach at 22.4·10$^3$ cm$^{-1}$. In black the difference between $H_2S_0^{2+}$ and $S_0$ ground states (inverse of black curve Figure 3c) obtained from steady-state absorption spectroscopy shown in Figure 1c. In green the reconstructed spectrum by adding the black curve to the orange spectrum after scaling it to the bleach, simulating how a transient spectrum would look like when the excited state is that of a $H_2T_1^{2+}$ but originating from a $S_0$ ground state molecule. The reconstructed spectrum is very similar to the spectrum in Figure S6c, indicating that $H_2T_1^{2+}$ is formed. (**b**) Comparison of the spectrum of the first state obtained from the global fitting procedure (dots) with one measured on TCPP in the absence of acid (line, Figure S2b), *i.e.* a $T_1$ differential spectrum. This indicates that the first species is neutral $T_1$. (**c**) Comparison of the spectrum of the second state obtained from the global fitting procedure (dots) with the green spectrum discussed in panel a (line), *i.e.* a $H_2T_1^{2+}$ differential spectrum after exciting a $S_0$ molecule. (**d**) Comparison of the spectrum of the third stated obtained from the global fitting procedure (dots) with the difference spectrum of $H_2S_0^{2+}$ and $S_0$ in the ground state (black line in panel a), *i.e.* a ground state differential spectrum after photo-induced protonation in the triplet state.
The reference spectra (lines) in panels b–d are scaled to match the spectra from the global fit (dots). The high level of similarity between the three spectra of the global fitting procedure and the separately obtained reference spectra proves that the excited state dynamics discussed in Figure 3 are correct.

## S2. Rate equation model for global fit

We consider three excited state levels like discussed in the main text. The rate equations of the states are the following:

$$\frac{\mathrm{d}N_T}{\mathrm{d}t} = -k_{d,T}N_T(t) - k_{p,T}N_T(t)$$

$$\frac{\mathrm{d}N_{HT}}{\mathrm{d}t} = k_{p,T}N_T(t) - k_{d,HT}N_{HT}(t)$$

$$\frac{\mathrm{d}N_{HS}}{\mathrm{d}t} = k_{d,HT}N_{HT}(t) - k_{dp,HS}N_{HS}(t).$$

These rate equations were solved using Mathematica under the conditions $N_T(0) = 1, N_{PT}(0) = 0,$ and $N_{PG}(0) = 0$. The subsequent result is used for the global fitting procedure.

## S3. Computational chemistry

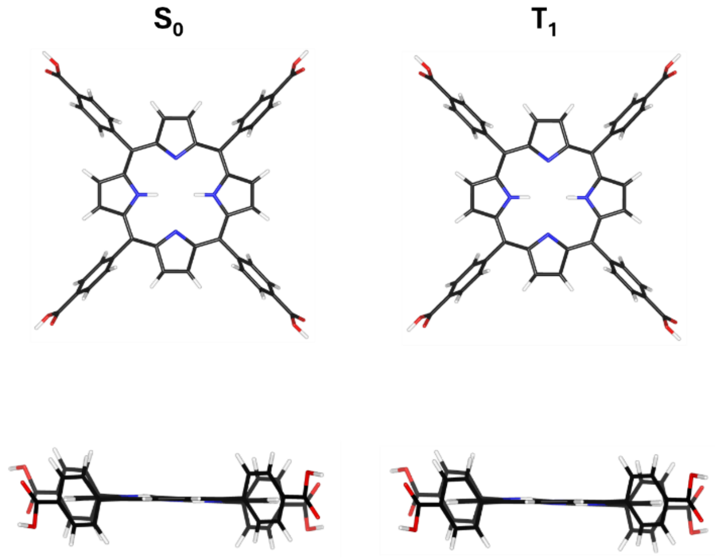


**Figure S7. Optimized geometries of the neutral ground state and triplet state $TCPP^0$.**

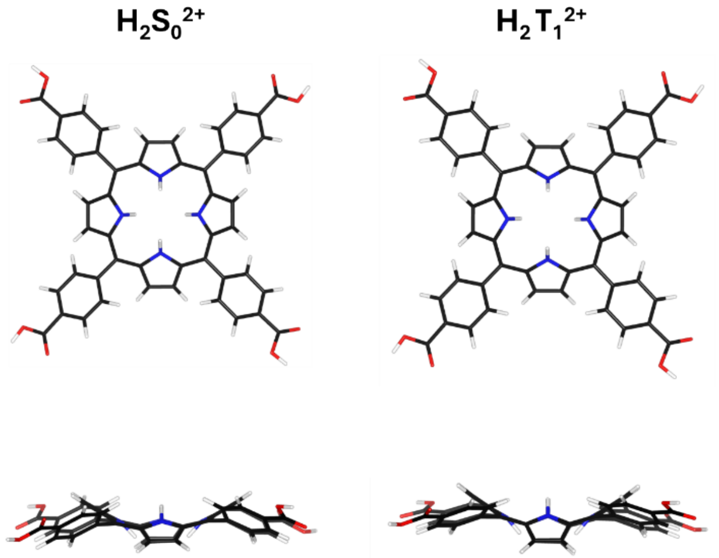


**Figure S8. Optimized geometries of the protonated ground state and triplet state $H_2TCPP^{2+}$.**

**Table S1: Results from geometry optimization and natural population analysis (NPA) for neutral and protonated $S_0$ and $T_1$.**

| **Structure** | **$S_0$ (TCPP$^0$)** | **$T_1$ (TCPP$^0$)** | **$S_0$ (TCPP$^{2+}$)** | **$T_1$ (TCPP$^{2+}$)** |
|---|---|---|---|---|
| N–H [Å] | 1.011, 1.011 | 1.010, 1.010 | 1.016, 1.016, 1.016, 1.016 | 1.016, 1.016, 1.017, 1.017 |
| **Dihedral angles [°]** | | | | |
| $N–C_p–C_p–N$ | 2.5 | 2.9 | 34 | 41 |
| $C_p–C_p–C_p–C_p$ | 1.9 | 2.4 | 42 | 48 |
| $C_m–C_m–C_m–C_m$ | 2.4 | 2.4 | 1.9 | 2.7 |
| **Charges (from NPA) [*e*]** | | | | |
| q(NH) | −0.481, −0.482 | −0.459, −0.459 | −0.499, −0.499, −0.500, −0.499 | −0.485, −0.496, −0.490, −0.500 |
| q(N) | −0.528, −0.529 | −0.482, −0.483 | n/a | n/a |
| q(H) | 0.463, 0.462 | 0.457, 0.457 | 0.421, 0.421, 0.421, 0.421 | 0.420, 0.423, 0.421, 0.419 |

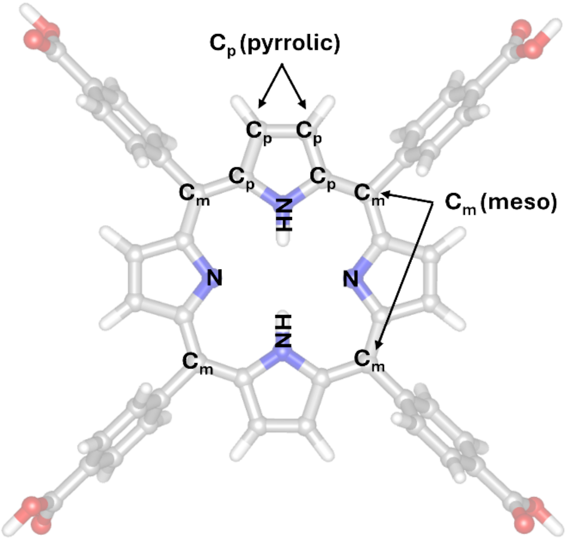


**Figure S9. Atom labels used in Table S1.**

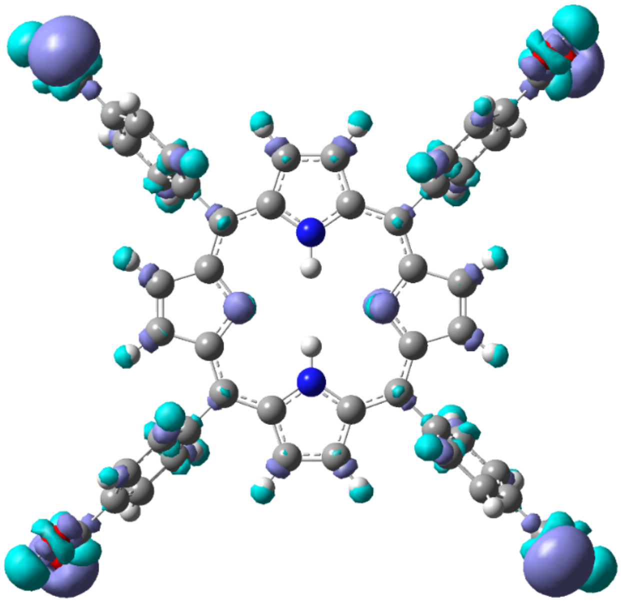

**Figure S10. Differential electron density plots for $T_1$ in the gas phase subtracted from the on for $T_1$ in DMF.** Violet: positive change; blue: negative change. Changes in electron density are mainly seen in the carboxylic acid groups.

## References


1 M. C. DeRosa and R. J. Crutchley, Photosensitized singlet oxygen and its applications, *Coord. Chem. Rev.*, 2002, **233–234**, 351–371.

2 T. Sato, Y. Hamada, M. Sumikawa, S. Araki and H. Yamamoto, Solubility of oxygen in organic solvents and calculation of the Hansen solubility parameters of oxygen, *Ind. Eng. Chem. Res.*, 2014, **53**, 19331–19337.
